# 

**Published:** 1887-10-08

**Authors:** 


CONTENTS.

An Experiment in Sociology - - - - - 19

Words of Consolation.—Chapter LIII. The Sufferings of Christ - - - - - - - - - 20

The Doctor's Pulpit.—Supper and Sleep - - - - 21

Our Hospitals as Patients Find Them - - - 22

Oliver Wendell Holmes. By Dr. CATERINA - - - 23

Abstract of Introductory Lecture delivered at St. Mary's Hospital. By ANDERSON CRITCHETT, M.A. 24

Professional Notes. By a PHYSICIAN - - - - 25

Notes and News.—Donations.—Hospital Appointments.—The Queen's Hospital, Birmingham.—The Hospitals Association Report and Notices.—New Hospital Subscriptions.—Vacancies.—The Sheffield Corporation and the Small-pox Hospital.—Working Class Hospital Collections.—The Charity Organisation Society.—Floral and Musical Service.—Dissenters and the Hospital Sunday Collections, etc., etc. - - - 26

Annotations.—Coroners: Legal or Medical?—Miss Twining on Village Life.—Ladies, Physic, and Mr. Ruskin - - 29

Medicine Men of the World. By the MAN IN THE CROWD - - - - - - - - 30

East and West. By LESLIE KEITH.—Chapter II. David and Mary Jardine - - - - - - - - - 31

Results of Fourth Quarterly Prize Competitions 33

The National Pension Fund - - - - - 33

The Editor's Letter-Box.—Hospital Statistics: Still More Details Wanted - - - - - - - - 33

Questions and Answers - - - - - - - 33

In- and Out-Patients' Hospital Diary.—General Hospitals - - - - - - - - - 34

Amusements with Prizes, for all Ages.—For Men and Women.—The Children's Corner - - - - - iv

**Hospitals urgently needing Annual Subscriptions, Donations, and Legacies.**

